# Fat Harvesting for Micro-Fragmented Adipose Tissue Injections: A Pilot Study Comparing Safety in Procedures Performed by Orthopedic and Plastic Surgeons

**DOI:** 10.1055/s-0045-1813003

**Published:** 2025-12-10

**Authors:** Bruno Butturi Varone, Rodrigo Bernstein Conde, Chilan B. G. Leite, Pedro Nogueira Giglio, Riccardo Gomes Gobbi, Marco Kawamura Demange

**Affiliations:** 1 Knee Group, Instituto de Ortopedia e Traumatologia, Hospital das Clínicas (HCFMUSP), Faculdade de Medicina, Universidade de São Paulo, São Paulo, SP, Brazil; 2 Instituto Universitario de Ciencia de la Salud Fundación H. A. Barceló, Facultad de Medicina, Buenos Aires, Argentina; 3 Department of Orthopedic Surgery, Brigham and Women's Hospital, Harvard Medical School, Boston, MA, United States

**Keywords:** adipose tissue, osteoarthritis, osteoarthritis, knee, osteoartrite, osteoartrite do joelho, tecido adiposo

## Abstract

**Objective:**

To compare short-term complication rates of small-volume adipose tissue harvesting for micro-fragmented adipose tissue (mFAT) knee injections performed by orthopedic surgeons with those performed by plastic surgeons. Additionally, to evaluate the orthopedic surgeon's learning curve.

**Methods:**

The present case-control study enrolled patients with knee osteoarthritis. All patients underwent a single-stage procedure consisting of abdominal adipose tissue harvesting, processing of the extracted material using the Lipogems (Lipogems International SpA) device to obtain mFAT, which was then injected intra-articularly into the knee. The patients were divided into a test group, with harvesting being performed by a recently trained orthopedic surgeon, and a control group, in which the procedure was performed by an experienced plastic surgeon. Short-term adverse effects, minor and major complications related to harvesting, were assessed intraoperatively and at 7-day follow-up.

**Results:**

No major complications (fat embolism, thromboembolic events, abdominal perforation, wound infection, dehiscence, or cosmetic changes) were observed in either group. Abdominal discomfort during harvesting, classified as a minor complication, showed no statistically significant difference between groups ( *p* = 0.362). Postoperative adverse effects, such as abdominal ecchymosis ( *p* = 0.362) and discomfort ( *p* = 0.342), were equivalent in both groups and resolved within 7 days.

**Conclusion:**

The present pilot study suggests that, with adequate training, orthopedic surgeons can perform small-volume adipose tissue harvesting with low complication rates, comparable to those achieved by plastic surgeons experienced in liposuction.

## Resumo

**Objetivo:**

Este estudo teve como objetivo comparar as taxas de complicações em curto prazo da coleta de tecido adiposo em pequeno volume para injeções de tecido adiposo microfragmentado (mFAT) no joelho realizada por um cirurgião ortopédico ou um cirurgião plástico, além de avaliar a curva de aprendizado de cirurgiões ortopédicos.

**Métodos:**

Este estudo de caso-controle incluiu pacientes com osteoartrite de joelho. Todos os pacientes foram submetidos a um procedimento em estágio único composto por coleta de tecido adiposo abdominal, processamento do material extraído com o dispositivo Lipogems (Lipogems International SpA) para obtenção de mFAT, que foi injetada via intra-articular no joelho. Os pacientes foram divididos em grupo teste, no qual a coleta foi realizada por um cirurgião ortopédico recém-treinado; e grupo controle, no qual o procedimento foi feito por um cirurgião plástico com experiência. Efeitos adversos em curto prazo e complicações mais e menos sérias relacionadas à coleta foram avaliados no período intraoperatório e à consulta de acompanhamento 7 dias após a cirurgia.

**Resultados:**

Nenhum paciente apresentou complicações mais sérias (embolia gordurosa, eventos tromboembólicos, perfuração abdominal, infecção da ferida, deiscência ou alterações estéticas). A taxa de desconforto abdominal durante a coleta, classificado como complicação menor, não apresentou diferença estatisticamente significativa entre os grupos ( *p* = 0,362). Os efeitos adversos pós-operatórios, como equimoses ( *p* = 0,362) e desconforto ( *p* = 0,342) abdominais, foram equivalentes em ambos os grupos e se resolveram em 7 dias.

**Conclusão:**

Este estudo piloto sugere que, com treinamento adequado, cirurgiões ortopédicos podem realizar a coleta de tecido adiposo em pequeno volume com baixas taxas de complicações, comparáveis às observadas em procedimentos realizados por cirurgiões plásticos experientes em lipoaspiração.

## Introduction

Knee osteoarthritis (KOA) is a highly prevalent condition that affects a significant share of the global population. The overall prevalence of symptomatic KOA is estimated at 30%, with rates reaching 44% in men and 28% in women aged 55 to 64 years. 1 Treatments vary according to symptom severity and patients' needs. Injectable therapies are widely used to provide intermediate-term pain relief. Among the available injectable options, micro-fragmented adipose tissue (mFAT) has gained attention for its promising results in pain reduction. 2 3 Several studies, including clinical trials, have reported superior outcomes in KOA patients treated with mFAT compared to those receiving conservative treatment or hyaluronic acid injections. 4 5 6 7 8

In aesthetic and reconstructive settings, liposuction is considered a minor procedure when the extracted volume is less than 1,000 mL. It is typically performed under local anesthesia, being associated with low complication rates. In contrast, large-volume liposuctions (i.e. > 10% of body weight) generally require general anesthesia and are associated with higher risks, including infection, hematoma, ecchymosis, and fat or pulmonary embolism. 9

However, in orthopedic practice, the volume of adipose tissue required is minimal. Studies suggested that harvesting less than 50 mL is sufficient to produce 5 to 10 mL of mFAT, an adequate volume for injections into large joints such as the knee. 10 11 Given the small volume and minimally invasive natures of the procedure, local anesthesia is usually sufficient—supporting the notion that, with proper training, orthopedic surgeons could be well suited to perform it safely.

While small-volume liposuction may be feasible for orthopedic surgeons, assessing its safety when performed by nonplastic surgery specialists remains essential. Therefore, this study aims to compare the short-term complication rates of small-volume adipose tissue harvesting for mFAT injections in KOA, when performed by orthopedic versus plastic surgeons. We hypothesize that complication rates are comparable between the two specialties.

## Methods

The present case-control study was approved by the local ethics committee (CAAE: 52440821.5.0000.0068), and all participants provided written informed consent. We assessed adverse effects and complications related to abdominal adipose tissue harvesting in patients with KOA. In the test group, the procedure was performed by a recently trained orthopedic surgeon and, in the control group, by a plastic surgeon. All patients underwent a single-stage procedure consisting of abdominal adipose tissue harvesting, processing with the single-use Lipogems device (Lipogems International SpA) to obtain mFAT, followed by intra-articular injection into the affected knee.

Patients were eligible to receive unilateral or bilateral intra-articular knee injections based on clinical symptoms. Inclusion criteria were patients with symptomatic KOA confirmed by radiographic evaluation, with all Kellgren-Lawrence grades (1–4) accepted. Exclusion criteria were varus or valgus deformity greater than 10° and body mass index (BMI) above 40kg/m ^2^ .

A total of 33 procedures were performed. In the first 13 cases, adipose tissue harvesting was conducted by a board-certified plastic surgeon with extensive liposuction experience, while a board-certified orthopedic surgeon observed and received hands-on training. The remaining 20 procedures were conducted independently by the orthopedic surgeon. All intra-articular knee injections were performed under ultrasound guidance.

### Adipose Tissue Harvesting and mFAT Processing

**Fig. 1:**
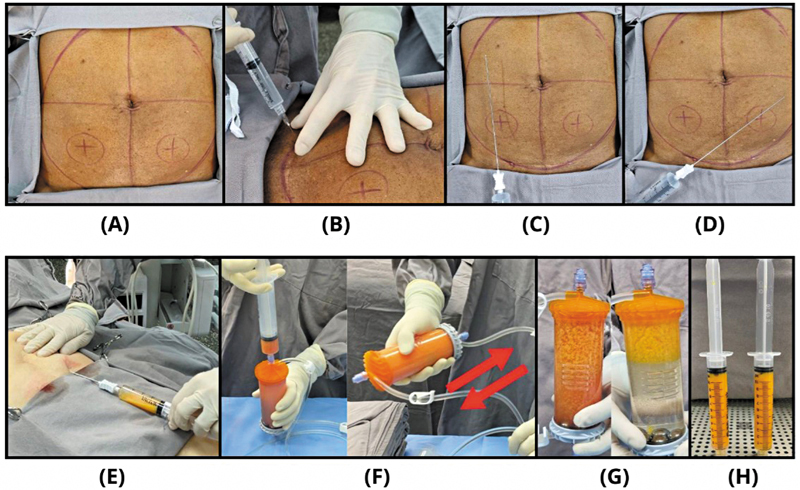
( **A** ) Skin portals were marked bilaterally above the inguinal line. ( **B** ) 1 mL of 2% lidocaine was administered at each portal. ( **C, D** ) Anesthetic solution was infiltrated into each hemi-abdomen. ( **E** ) Adipose tissue was harvested using a 13G cannula connected to a VacLock syringe. ( **F** ) Mechanical microfragmentation of the harvested adipose tissue. ( **G** ) Changes in tissue appearance following successive washes and fragmentation. ( **H** ) Final mFAT product ready for intra-articular injection into the knee.

Abdominal adipose tissue harvesting, processing with the Lipogems device, and ultrasound-guided injections were performed in a single-stage procedure. Patients were placed in a supine position under sterile conditions in the operating room and received local anesthesia. The lower abdomen was chosen as the standardized harvest site due to accessibility and consistent adipose tissue volume. Skin portals were marked bilaterally above the inguinal line ( Fig. 1A ), and 1 mL of 2% lidocaine was administered at each portal ( Fig. 1B ). After allowing time for anesthetic onset, small incisions of approximately 4 mm were made using an 11-blade scalpel.

Adipose tissue was harvested using a two-stage tumescent technique. 12 Anesthetic infiltration involved introducing a cannula through each portal to distribute the solution throughout the subcutaneous harvest area. Infiltration was performed using a 19G cannula provided in the Lipogems kit. The injected anesthetic solution was composed of 20 mL of 2% lidocaine, 20 mL of 0.5% bupivacaine, 1 mL of 1 mg/mL adrenaline, and 250 mL of 0.9% saline, yielding a total volume of 291 mL. This total was split into 120 mL for each hemi-abdomen, with the remaining 51 mL reserved for additional anesthesia if necessary ( Figs. 1C and D ). Following an appropriate latency period, adipose tissue was harvested using a 13G cannula (also provided in the Lipogems kit) connected to a VacLock syringe (Merit Medical Systems Inc.), as shown in Fig. 1E . Harvesting was performed uniformly, avoiding repeated extraction near the portal sites to minimize potential cosmetic concerns.

The remaining subcutaneous fat was assessed by repeatedly pinching the area, a reliable technique to ensure an adequate tissue layer remained and to avoid cosmetic alterations. 13 For patients with unilateral KOA, 60 mL of adipose tissue was harvested, while those with bilateral underwent harvesting of 120 mL. These volumes were standardized based on previous studies indicating the amount of adipose tissue required to yield 10 mL of mFAT per knee. 14

After harvesting, the skin portals were closed using 5.0 nylon sutures. Adipose tissue processing was performed with the single-use Lipogems device, following protocols established by previous studies ( Figs. 1F–H ). 2 The resulting mFAT was injected into the knee via a superolateral approach under ultrasound guidance using a 16G needle. Immobilization was not required, and patients were encouraged to begin knee range of motion exercises on the first day after the procedure.

### Data Collection

Demographic data, including age, sex and BMI were collected from each patient. The volume of adipose tissue harvested and the amount of mFAT obtained were also recorded. Complications related to the harvesting procedure were assessed on the same day and again at a scheduled 7-day follow-up visit. Complications were categorized according to the Clavien-Dindo system, as follows:

- Major complications (Clavien-Dindo grade III or higher) included fat embolism, venous thromboembolic events, abdominal perforation, wound infection requiring surgical intervention, intra-articular infection, and significant cosmetic alterations.
- Minor complications (Clavien-Dindo grades I and II) included expected or mild adverse events such as abdominal ecchymosis, discomfort, or other complications resulting in no residual impairment.

### Statistical Analysis

Categorical variables were reported as the number of events and corresponding percentages, while continuous variables were presented as mean ± standard deviations (SDs). The Shapiro-Wilk test was used to assess the normality of continuous variables. Depending on distribution, comparisons between continuous variables were made using either the t-test or Mann-Whitney test. Fisher's exact test was utilized for comparing categorical variables. Statistical significance was defined as *p* -value < 0.05.

## Results

A total of 33 patients were included in the study. Of those, 13 underwent adipose tissue harvesting by a plastic surgeon, and 20 by an orthopedic surgeon, divided into groups. The mean age was 61.2 ± 10.2 years in the plastic surgeon group, and 60.4 ± 8.6 years in the orthopedic surgeon group ( *p* = 0.802). The mean BMI was 27.7 ± 4.5 kg/m ^2^ in the plastic surgeon group and 29.3 ± 4.6 kg/m ^2^ in the orthopedic one ( *p* = 0.349).

The mean volume of adipose tissue harvested did not differ significantly between groups (plastic: 117.7 ± 15.4 mL vs. orthopedic: 123.0 ± 33.8 mL; *p* = 0.245). Similarly, the volume of mFAT obtained after processing the adipose tissue was comparable between the groups (28.5 ± 3.1 mL vs. 27.4 ± 11.1 mL; *p* = 0.645).

No major complications were observed in either group. There were two minor complications—described as patient discomfort during the harvesting procedure—reported in each group. At the 7-day follow-up, abdominal ecchymosis was observed in 12 patients (92.3%) in the plastic surgeon group and 17 (85.0%) in the orthopedic one. Abdominal discomfort was reported in 11 (84.6%) in the plastic surgeon group and 16 (80.0%) in the orthopedic surgeon group. Baseline characteristics and outcomes are summarized in Table 1 .

**Table 1:** Patient characteristics and adverse events according to surgeon specialty

	Orthopedic surgeon	Plastic surgeon	*p-* value
Number of cases	20	13	
Age, years	60.4 ± 8.6	61.2 ± 10.2	0.802
Sex, female/male	17/3	10/3	0.659
BMI, kg/m ^2^	29.3 ± 4.6	27.7 ± 4.5	0.349
Bilateral KOA	12 (60)	11 (84.6)	0.246
Adipose tissue volume, ml	123.0 ± 33.8	117.7 ± 15.4	0.245
mFAT volume, ml	27.4 ± 11.1	28.5 ± 3.1	0.645
Minor complications	2 (18.2)	2 (10)	0.362
Abdominal ecchymosis	17	12	0.362
Abdominal discomfort	16	11	0.342

**Abbreviations:** BMI, body mass index; KOA, knee osteoarthritis; mFAT, micro-fragmented adipose tissue.

**Note:** Values are expressed as mean ± SD or number (percentage).

## Discussion

The main finding of this pilot study is that, with proper training, orthopedic surgeons can safely and effectively perform adipose tissue harvesting for mFAT injections. There were similarly low complication rates in procedures performed by orthopedic and plastic surgeons. Additionally, the similar volumes of adipose tissue harvested and processed in both groups support the feasibility of this procedure when performed by orthopedic surgeons.

Notably, the average volume of mFAT obtained per patient was nearly three times greater than the amount typically required for a single large-joint injection, such as in the knee. This is particularly relevant for patients undergoing unilateral injections, for whom smaller harvest volumes may suffice.

Previous studies have reported low complication rates for liposuction, even with larger volumes. For instance, Aljerian et al. 15 conducted a meta-analysis including 60 studies and 21,776 patients, reporting an overall complication rate of 12%, primarily due to ecchymosis and edema. When these side effects were excluded, the rate of minor complications dropped to 5%, and major complications were observed in only 1% of cases. Minor complications included cosmetic changes, seroma, hematoma, ecchymosis, and edema, while major complications encompassed deep venous thrombosis, sepsis, visceral damage, hypovolemia, and pulmonary complications. It is important to note that their analysis focused on high-volume liposuction procedures for aesthetic purposes.

Chow et al. 16 investigated thresholds for liposuction safety and concluded that exceeding 100 mL per unit of BMI may increase risk of complications. Consequently, in a patient with a BMI of 30 kg/m ^2^ , volumes of up to 3,000 mL were still considered safe. Notably, those procedures were performed under general anesthesia by board-certified plastic surgeons.

Another important consideration involves the potential risk of anesthetic toxicity. The literature suggests the safe use of lidocaine up to 35 mg/kg and, despite the relatively high dose used in tumescent techniques, this approach remains considered safe for several reasons. 17 First, although the technique requires a large volume of dilute anesthetic solution, the addition of epinephrine slows lidocaine absorption, with peak plasma levels typically occurring 12 to 14 hours after the infiltration. Furthermore, liposuction itself reduces the total amount of lidocaine absorbed systemically. The technique also minimizes blood loss due to the vasoconstrictor effects of epinephrine. Taking these factors into account, we chose to employ the tumescent technique, which proved effective in minimizing abdominal discomfort during adipose tissue harvesting.

Regarding the injected volume, in our study, 10 mL of mFAT was injected in each affected knee. While this volume appears appropriate for large joints, smaller ones may require even less. In a case series of patients with hip osteoarthritis, Dall'Oca et al. 18 reported harvesting 60 mL of adipose tissue, which yielded 5 to 10 mL of mFAT; this amount effectively reduced pain and improved clinical outcomes. Similarly, D'Ambrosi et al. 19 harvested 45 mL of adipose tissue and injected 5 mL of mFAT into arthritic ankles, also with favorable clinical results. These examples highlight that orthopedic applications typically require smaller volumes than for cosmetic purposes, even for bilateral procedures involving large joints. This distinction is important, as it supports the feasibility of performing such procedures in outpatients' settings—an especially relevant point given the high prevalence of osteoarthritis and the importance of cost containment. Therefore, by reducing hospitalization, operating room time, and anesthesia costs, this approach may broaden access to regenerative treatments.

In our study, patient discomfort during the procedure—including inadequate positioning or harvesting-related—was classified as a minor complication. Because all procedures were performed under local anesthesia, these events were more frequent during the early learning curve. However, as both the orthopedic and plastic surgeons gained experience, such occurrences became less common. We attribute this initial discomfort to a slower procedural pace in the first cases. This highlighted the importance of optimal and ergonomic patient positioning, particularly under local anesthesia. Additionally, careful mapping of the harvesting area and complete infiltration with the tumescent solution appeared important for minimizing discomfort during the harvesting. Although we prepared 291 mL of Klein's solution for local anesthesia, only 240 mL were administered (120 mL per hemiabdomen). The remaining volume was reserved for use if additional anesthesia became necessary.

No major complications were observed in our pilot study. Other studies assessing the safety of mFAT for KOA have reported similarly low complication rates related to adipose tissue harvesting. 20 21 22 23 Likewise, Aljerian et al. estimated major complication rates as low as 1%, even in large-volume liposuction procedures. 15

Visceral and vascular injuries caused by infiltration needles or liposuction cannulas are rare and have mostly been reported in patients with abdominal scars or hernias, or in high-volume cases performed under general anesthesia. 24 25 26 In our study, all patients underwent thorough preoperative evaluation, and previous abdominal incisions were avoided by employing the two-portal technique. Furthermore, abdominal discomfort and ecchymoses were anticipated as common side effects. These events were discussed with all patients during the consent process, and topical or pain medications were used to attenuate ecchymosis or abdominal discomfort.

Our study is not without limitations. The plastic surgeon performed the initial 13 procedures, while the subsequent 20 were carried out by the orthopedic surgeon. As such, it is plausible that the surgical team gained familiarity with the protocol over time, potentially improving efficiency in the later cases. However, it is important to note that the plastic surgeon already had prior experience with liposuction in other clinical contexts. In contrast, the orthopedic surgeon had no prior hands-on experience and only limited observational training during the initial cases, making it unlikely that this brief exposure surpassed the expertise of the plastic surgeon.

Another limitation is the absence of a formal sample size calculation, which, although acceptable for a pilot study, limits the generalizability of the findings. Therefore, randomized clinical trials specifically designed to compare outcomes of adipose tissue harvesting between surgical specialties could provide more robust evidence. Nevertheless, to our knowledge, this is the first study to compare complication rates between orthopedic and plastic surgeons. Our findings contribute to the literature by showing that small-volume adipose tissue harvesting can be safely performed by trained orthopedic surgeons.

## Conclusions

Assessing the safety of small-volume adipose tissue harvesting performed by orthopedic surgeons is essential to broaden access to this treatment. Our findings support the notion that, with appropriate training, orthopedic surgeons can achieve low complication rates when harvesting limited volumes of adipose tissue for orthopedic applications. As an exploratory study, these results provide a foundation for future research aimed at evaluating the learning curve and comparing outcomes across different harvesting sites.
